# From local uncertainty to global predictions: Making predictions on fractal basins

**DOI:** 10.1371/journal.pone.0194926

**Published:** 2018-04-18

**Authors:** Asaf Levi, Juan Sabuco, Michael Small, Miguel A. F. Sanjuán

**Affiliations:** 1 Nonlinear Dynamics, Chaos and Complex Systems Group, Departamento de Física, Universidad Rey Juan Carlos, Madrid, Spain; 2 Institute for New Economic Thinking at the Oxford Martin School, Mathematical Institute, University of Oxford, Oxford, United Kingdom; 3 School of Mathematics and Statistics, University of Western Australia, Crawley, Australia; 4 Department of Applied Informatics, Kaunas University of Technology, Kaunas, Lithuania; 5 Institute for Physical Science and Technology, University of Maryland, College Park, Maryland, United States of America; University of Campinas, BRAZIL

## Abstract

In nonlinear systems long term dynamics is governed by the attractors present in phase space. The presence of a chaotic saddle gives rise to basins of attraction with fractal boundaries and sometimes even to Wada boundaries. These two phenomena involve extreme difficulties in the prediction of the future state of the system. However, we show here that it is possible to make statistical predictions even if we do not have any previous knowledge of the initial conditions or the time series of the system until it reaches its final state. In this work, we develop a general method to make statistical predictions in systems with fractal basins. In particular, we have applied this new method to the Duffing oscillator for a choice of parameters where the system possesses the Wada property. We have computed the statistical properties of the Duffing oscillator for different phase space resolutions, to obtain information about the global dynamics of the system. The key idea is that the fraction of initial conditions that evolve towards each attractor is scale free—which we illustrate numerically. We have also shown numerically how having partial information about the initial conditions of the system does not improve in general the predictions in the Wada regions.

## Introduction

Predicting the future state of a nonlinear dynamical system may be very challenging. Recently the use of sophisticated prediction techniques, like neural networks, has allowed researchers to improve the prediction ability in such systems [1]. But this type of methods cannot be always easily applied. In many nonlinear dynamical systems, complex structures arise and change their shape within phase space as one parameter is varied. Basins of attraction are an interesting example of these structures in dissipative and Hamiltonian systems. Roughly speaking, we can say that a basin of attraction is the set of initial conditions that evolve in time towards a given attractor. In many nonlinear systems there are several attractors coexisting in phase space, which can have fractal boundaries separating their basins. This fact can make the study of the global dynamics and the predictability of the system a very difficult task. Nonlinear systems with fractal basins can be classified basically in four different categories: intertwinned basins, Wada basins, riddled basins and sporadically fractal basins [2]. When a dynamical system possesses this kind of basins it is very difficult to make predictions, due to the fact that there is an intrinsic uncertainty on the final state of a given initial condition taken in the neighborhood of the fractal boundary. The physical reason behind this is the finite accuracy in the measurement of the initial conditions for any real system. Furthermore, in systems with fractal basins there are infinitely many close points that can go to a different attractor. The situation gets even more complicated if we do not have access to the time series of the dynamical system and the only observables of the system are the attractors.

**Fig 1:**
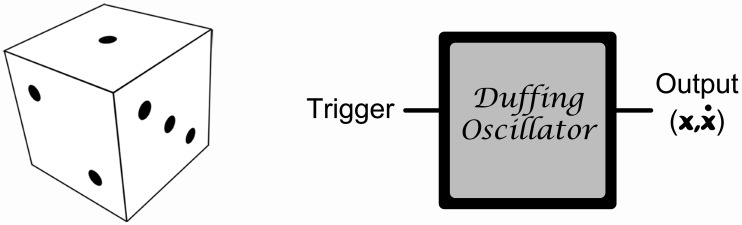
Black box diagram. We consider that the dynamical system that we are going to study is a black box to which we do not have any internal access. We can only measure the final state of the system for a given initial condition. In this sense, the problem that we face is very similar to the problem of predicting the final state of a die.

Although the problem is far from being solved, recently two useful ideas proposed by Menck *et al*. and Daza *et al*. namely basin stability [3] and basin entropy [4] have shed some light on important properties of complex basin structures. Here, we present a general procedure to provide some kind of statistical prediction in nonlinear systems with fractal basins, where the only observables that we have access to are the attractors of the system. We assume that we are not able to measure the time series before they reach the final attractor, but we assume that we have some knowledge about the probability density function of the initial conditions. In this way, we consider that the dynamical system is like a black box, as depicted in Fig 1, where only the final output can be measured. In this framework, the behavior of the dynamical system is very similar to that of a die, although the behavior of this one is neither chaotic nor random [5]. The key point of the prediction mechanism developed here, is (as we show in several ways) that the ratio or probability of initial conditions going to each attractor in the phase space is scale free. This is precisely what allows the statistical prediction. We show here how this procedure works for Wada basins, but it should also work for systems showing any of the other kind of fractal basins.

A dynamical system has Wada basins if it has three or more basins sharing the same fractal boundary. This topological idea was introduced by Kennedy and Yorke [6]. Wada basins usually appear in two-dimensional dynamical systems as a result of a boundary crisis of a chaotic attractor. This fact often leads to the fractalization of the entire basin boundary. Wada basin boundaries are frequently observed in both dissipative and Hamiltonian systems. We can find this topological property in relation to mechanical models of billiard balls [7] or chaotic advection of fluid flow [8] and in the context of the Hénon-Heiles Hamiltonian system in celestial mechanics [9]. Due to the structural complexity of the Wada basin boundaries, in practice, these structures imply serious problems in the long term prediction of dynamical systems, also known as final state sensitivity [10, 11].

Here, we study the Duffing oscillator for a choice of parameters that verifies the Wada property, based on the work of Aguirre and Sanjuan [12]. The Duffing oscillator is one of the best known models of nonlinear oscillators, with applications in many fields of applied sciences and engineering. The structure of the paper is as follows. Section 2 is devoted to the description of the Duffing oscillator and the methodology used to explore its phase space. The one-dimensional analysis of the model is described in Section 3. The two-dimensional analysis is done in Section 4. The implication of fractal boundaries on the probabilities of each basin of attraction is given in Section 5. Finally, some conclusions are drawn in the last section.

## Description of the Duffing oscillator

We consider here the periodically driven Duffing oscillator [13] that is described by the following differential Eq (1),

$$\ddot{x}+0.15\dot{x}-x+x^{3}=0.245\text{cos}\left(\omega t\right).$$ (1)

The Duffing oscillator of Eq (1) has a transient chaotic behavior and there are three coexisting periodic attractors whose basins of attraction have Wada boundaries [12]. We have used the stroboscopic map with *T* = 2*π* associated with the Duffing oscillator to compute the position of the attractors in phase space. We define as *P*1*R* and *P*1*L* the period-1 attractors located on the right and on the left, respectively. We define as *P*3*L*, *P*3*C* and *P*3*R* the points belonging to the period-3 attractor. The period-1 attractors are located at *P*1*R* ≈ (0.815, 0.242) and *P*1*L* ≈ (−0.933, 0.299). The period-3 attractor is located at *P*3*L* ≈ (−1.412, −0.137), *P*3*C* ≈ (−0.354, −0.614), and *P*3*R* ≈ (0.645, −0.464) [12]. The frequency is a critical parameter in the study of nonlinear oscillators [14, 15]. But this parameter is not so important in chaotic systems, since they have a broad spectrum which covers a wide range of frequencies [16].

**Fig 2:**
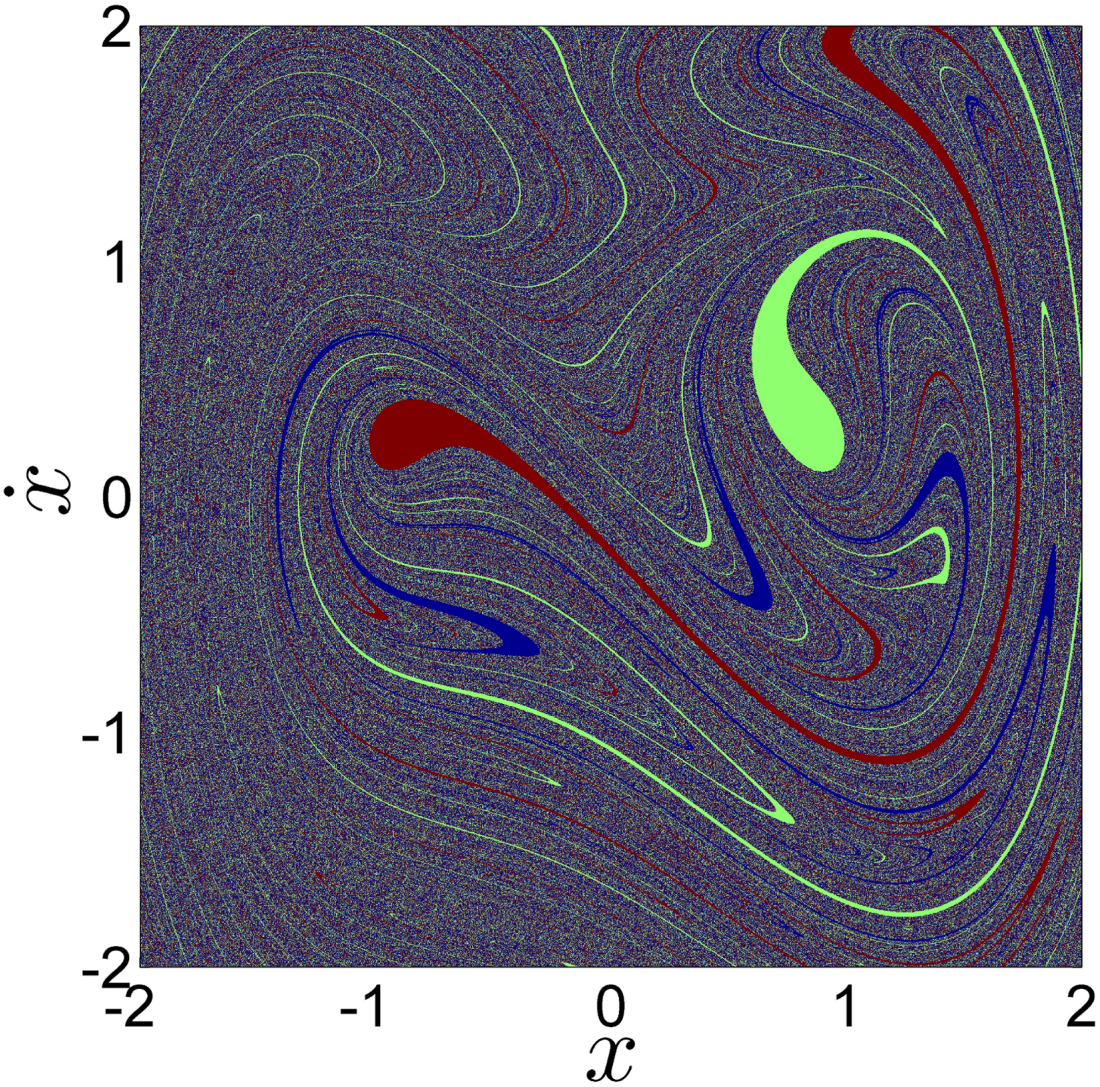
The basins of attraction of the Duffing oscillator of Eq 1. A fine grid of 2000 × 2000 of initial points is considered and different colors are chosen according to which attractor an initial condition goes to. The points that end up in the *P*3 attractor are colored in blue. The points that goes to the P1R attractor are colored in green. The initial conditions which final state is *P*1*L* are colored in red.

To compute the basins of attraction, we have taken all the initial conditions inside the square [−2, 2] × [−2, 2] of the phase space, and we have integrated the system using a fourth-order Runge-Kutta integrator with a fix integration step of 2*π*/4 × 10^5^, until their orbits reach the corresponding attractor. Different colors have been chosen according to which attractor an initial condition goes to, as shown in Fig 2. Every initial condition belonging to the basins of attraction of the period-1 attractors *P*1*L* and *P*1*R* have been plotted in red and green respectively. All the initial conditions belonging to the basin of attraction of the period-3 attractor have been plotted in blue. The phase space resolution depends on the amount of points taken in the horizontal and vertical axes. More points imply more resolution. For example, if we divide $\dot{x}$ and *x* in 400, we obtain a 400 × 400 matrix with 160, 000 initial conditions, where every initial condition has a two decimal precision. In the next sections we have studied the following matrices of initial conditions: 50 × 50, 100 × 100, 200 × 200, 300 × 300, 400 × 400, 500 × 500, 1000 × 1000, 2000 × 2000 and 3000 × 3000. We have started with a matrix of 2, 500 (50 × 50) initial conditions and finished with a matrix of 9 × 10^6^ (3000 × 3000) initial conditions.

To find the probability of reaching a given state in a dynamical system it is necessary to know its final probability density function (invariant measure). The evolution of an arbitrary probability density function in a dynamical system *f* is described by the Perron-Frobenius operator.

$$\rho_{n+1}\left(x\right)=L_{PF}\rho_{n}\left(x\right),$$ (2)

where *ρ*_*n*_ is the natural invariant after the *n* − *th* iteration of the map. The operator can be explicitly written as,

$$L_{PF}\rho_{n}\left(x\right)=\int \rho_{n}\left(x\right)\delta \left(x-f\left(y\right)\right)dy.$$ (3)

When only a finite number of non-chaotic attractors can be found in phase space, the evolution of the probability density function described by the Perron-Frobenius operator converges to delta functions. With knowledge of its invariant measure it is possible to determine the probability of ending on each attractor. However, in our case it is very difficult to use this analytical approach since we do not know the explicit expression of the time-2*π* map of the Duffing oscillator. For that reason, in the following sections we have used a much more quantitative procedure to compute the probabilities of the final state of the system. We have done this by directly sampling the entire phase space with a uniform grid of initial conditions, and computing the ratio of the number of points (of those initial conditions) ending in a particular final state relative to the number of the sample. Interestingly, we have found that this method works even for very low resolution samples.

We have divided the statistical analysis of the model into two parts. First, we have studied the probabilities obtained by sampling the phase space along horizontal (or vertical) one-dimensional straight lines. Second, we have used a two-dimensional grid covering the whole phase space to compute the probabilities associated with each attractor in phase space.

## One-dimensional analysis of the model

Our goal in the first analysis is to compute the probabilities of ending on a given attractor, assuming that we know only one of the two coordinates of the initial condition, either *x* or $\dot{x}$. This means that we need to compute a conditional probability. We could do this formally by taking as our initial probability density function *ρ*_0_ the one that is zero everywhere except in the coordinate that we know, and recursively applying the Perron-Frobenius operator until it converges. We then integrate the resulting probability density function in the neighborhood of each attractor to find the conditional probabilities

$$P\left(P1L|x=x_{i}\right),\;P\left(P1R|x=x_{i}\right),\;P\left(P3|x=x_{i}\right).$$ (4)

These are the conditional probabilities of ending up in each attractor given that we know the coordinate *x*_*i*_ of the initial condition. However, as we have already mentioned, finding the final probability density function using the Perron-Frobenius operator is usually very complicated.

**Fig 3:**
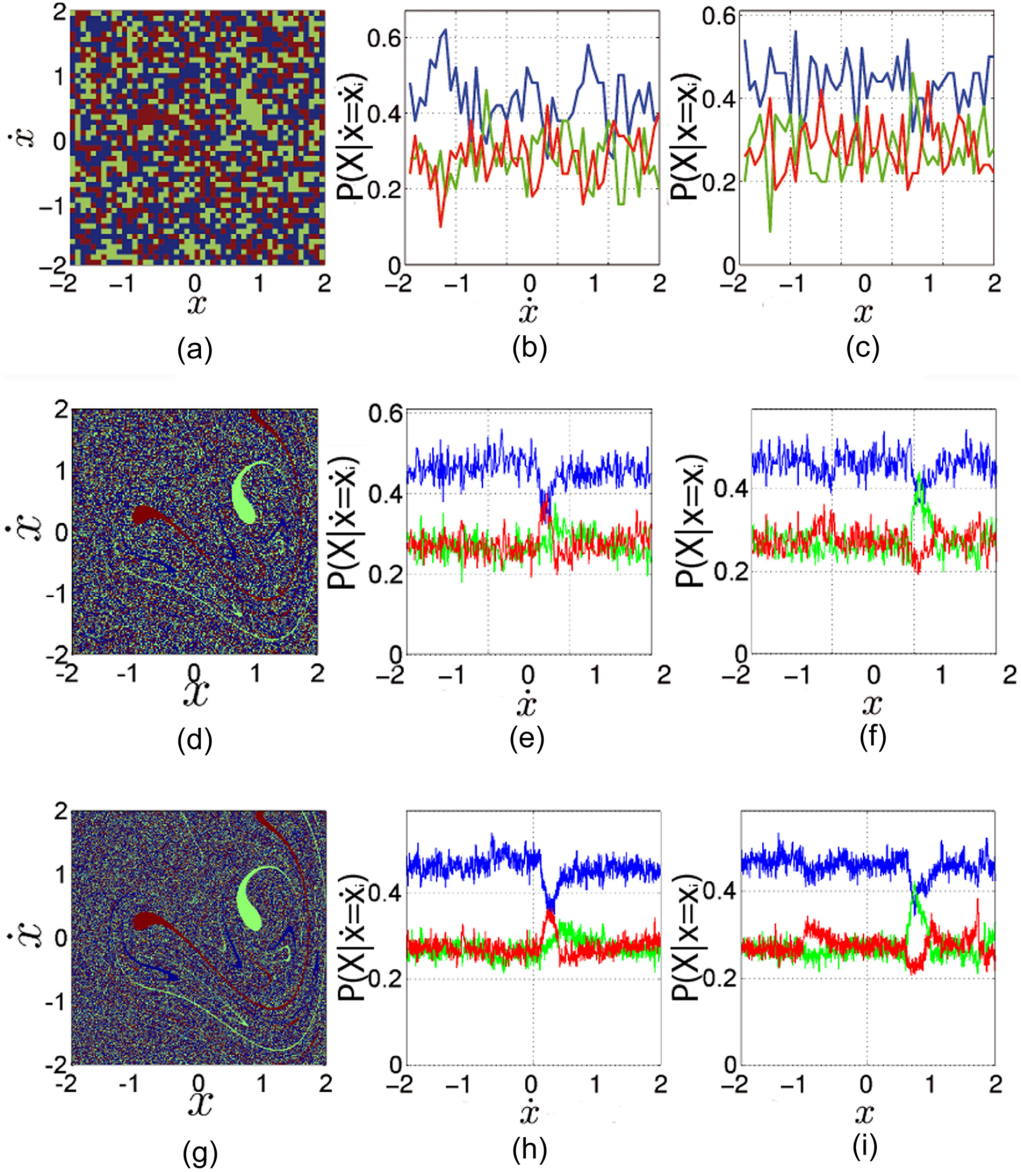
Conditional probabilities of each attractor in the Duffing oscillator for different resolution grids. Panels (a), (d) and (g) are the basins of attraction of the Duffing oscillator for grids of 50 × 50, 300 × 300 and 1000 × 1000 correspondingly. The points are colored according to which attractor an initial condition goes to. In panels (b), (e) and (h) we have plotted the conditional probabilities associated with each vertical line of the phase space, $P(P1L|\dot{x}={\dot{x}}_{i})$ (red), $P(P1R|\dot{x}={\dot{x}}_{i})$ (green) and $P(P3|\dot{x}={\dot{x}}_{i})$ (blue). In panels (c), (f) and (i) we have plotted the conditional probabilities associated with each horizontal line of the phase space, *P*(*P*1*L*|*x* = *x*_*i*_) (red), *P*(*P*1*R*|*x* = *x*_*i*_) (green) and *P*(*P*3|*x* = *x*_*i*_) (blue).

An alternative way to compute these conditional probabilities is to sample the phase space in *x*_*i*_ along $\dot{x}$ with a uniform one-dimensional grid, and then compute the final state of all of those initial conditions. Then taking the ratio of initial conditions that belongs to each basin of attraction gives us *P*(*P*1*L*|*x* = *x*_*i*_), *P*(*P*1*R*|*x* = *x*_*i*_) and *P*(*P*3|*x* = *x*_*i*_). We have done this for every *x*_*i*_ in different resolutions. We have followed a similar procedure to compute the conditional probabilities $P(P1L|\dot{x}={\dot{x}}_{i})$, $P(P1R|\dot{x}={\dot{x}}_{i})$ and $P(P3|\dot{x}={\dot{x}}_{i})$, where we assume that we know the coordinate ${\dot{x}}_{i}$ of the initial condition. We summarize the results in the diagrams and graphs shown in Fig 3.

As we can see in Fig 3, for the resolution 300 × 300 and higher, the conditional probabilities remain constant for almost every *x*_*i*_. It is also clear in Fig 3 that the period-3 attractor (blue basin) is the most probable attractor and the two period-1 attractors have almost the same probability (around 0.25). For the interval $\dot{x}=\left[0,0.5\right]$, a big change in the trend occurs, when $P(P3|\dot{x}={\dot{x}}_{i})$ (blue) loses over 24% of its value and $P(P1L|\dot{x}={\dot{x}}_{i})$ (red) sums 33% to its value. In this interval, the *P*1*L* attractor (red) is the most common, additionally $P(P1R|\dot{x}={\dot{x}}_{i})$ (green) sums 20% too and becomes more frequent inside this interval. The location of the two large basin cells of the period-1 attractors in phase space lies inside this interval, which explains the new trend of probabilities. In the interval *x* = [0.8, 1] another big change in the trend occurs when *P*(*P*3|*x* = *x*_*i*_) (blue) loses about 25% of its value and *P*(*P*1*R*|*x* = *x*_*i*_) (green) attractor increases its value by 55% of its value and becomes the most frequent attractor in phase space. Additionally, in the interval *x* = [1.3, 1.6] there is a peak in *P*(*P*1*L*|*x* = *x*_*i*_) (red). This attractor sums over 40% of its value and becomes the most common attractor in this small interval. Again this result arises from the location of the basin cells of the period-1 attractor in phase space. However, despite of those big local changes of the conditional probabilities near to the large basin cells, we find that in the rows or columns with a strong Wada property (which are the most common) the conditional probabilities are almost constant. The conditional probabilities only change in the regions with big basin cells.

**Fig 4:**
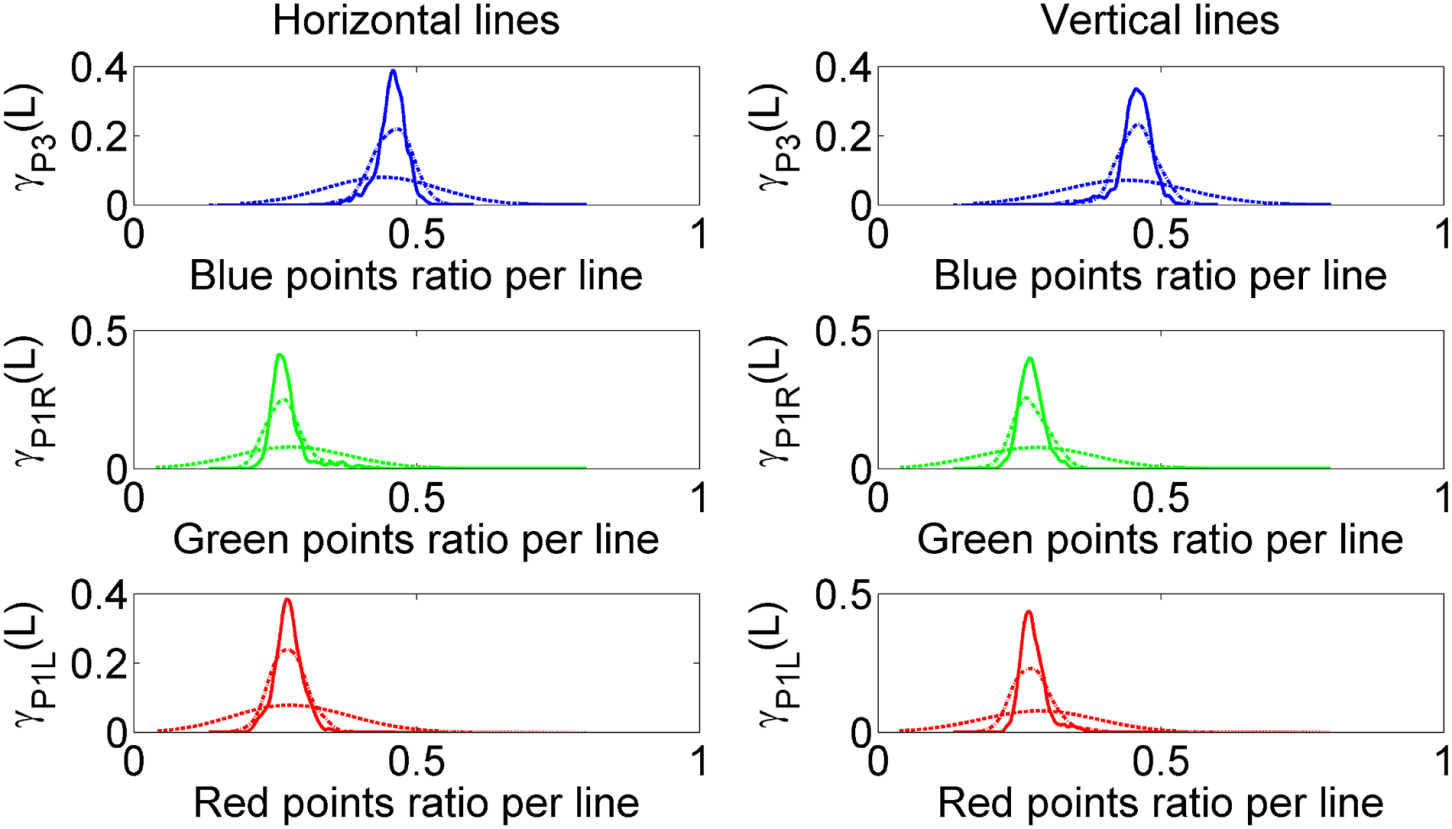
Kernel probability density estimation (KPDE) of the points ratio associated with each attractor for an horizontal or a vertical line. This figure shows the KPDE related to each attractor for a random picked horizontal or vertical line. The horizontal axis measures the ratio points per line. The vertical axis represents the probability density of each attractor, for horizontal lines on the left and for vertical lines on the right. Here, *γ*_*P*3_ (blue) is the probability density function associated with the *P*3 attractor, *γ*_*P*1*R*_ (green) is the probability density function associated with the *P*1*R* attractor and *γ*_*P*1*L*_ (red) is the probability density function associated with the *P*1*L* attractor. We have repeated the same computation for the following resolutions: 50 × 50 as dash line, 300 × 300 as dot dash line and 1000 × 1000 as solid line.

We can also treat the total length (found in a given horizontal or vertical straight line in the phase space) associated to the basin with each attractor, as a continuous random variable which will have associated a probability density function (pdf), *γ*(*L*). These pdfs allow us to compute the probability that the length of each attractor of a horizontal (or vertical) straight line (in phase space) is within a Δ*δ* interval, this is *P*(*L* − Δ*δ* < *L* < *L* + Δ*δ*). We have obtained the pdfs by counting the number of initial conditions for every one-dimensional horizontal (or vertical) straight line that goes to each attractor, and computing later the histogram of the number of horizontal (or vertical) lines vs the number of initial conditions. Normalizing this histogram by the number of horizontal (or vertical) lines we obtain the desired pdf. We can see the results of the computed pdfs for different resolutions in Fig 4. The length associated with each attractor in every straight line is measured by the number of initial conditions. In order to compare the different resolutions, we have also normalized the horizontal axes where we represent the points ratio per line. As we can see, as the phase space resolution increases the pdf shapes become smoother. On the one hand, from the statistical coefficients calculated from the data, we can conclude that the pdf associated with the length of the *P*3 attractor in either vertical or horizontal straight lines is not normally distributed and has a long tail in the left side. On the other hand the pdfs associated with the lengths in horizontal (or vertical) straight lines for the *P*1*L* and *P*1*R* attractors, are not normally distributed either, and have a long tail on the right side. As expected the mean of the pdf associated with the *P*3 attractor doubles the mean of the *P*1*L* and *P*1*R* attractors, either in the horizontal or the vertical direction. Interestingly, the standard deviation is about the same for all the pdfs—in both the horizontal and vertical directions.

## Two-dimensional analysis of the model

In the two-dimensional case the invariant probability density function would be computed taking as our initial probability density function *ρ*_0_, the one that is one everywhere in the square [−2, 2][−2, 2], and applying recursively the Perron-Frobenius operator until it converged. We would integrate the resulting probability density function in the neighborhood of each attractor to find the total probabilities,

P(P1L),P(P1R),P(P3). (5)

These are the total probabilities of ending in each attractor assuming that we do not know any of the coordinates of the initial conditions. However, as in the previous case, to find the final probability density function using the Perron-Frobenius operator is usually difficult.

**Fig 5:**
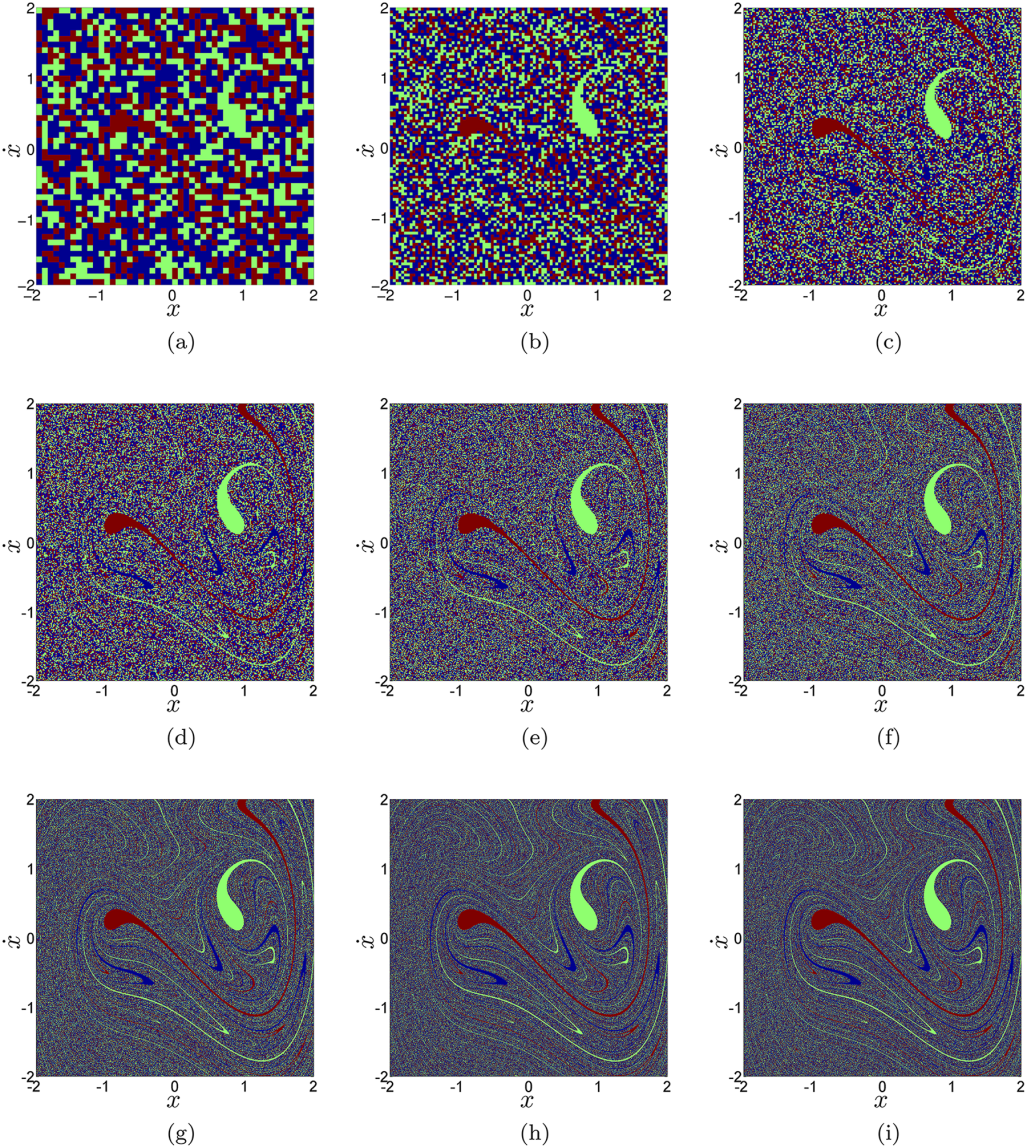
Basins of attraction for different resolutions. The picture shows the basin of attraction plotting, each one with different resolution. (a) 50 × 50, (b) 100 × 100, (c) 200 × 200, (d) 300 × 300, (e) 400 × 400, (f) 500 × 500, (g) 1000 × 1000, (h) 2000 × 2000 and (i) 3000 × 3000.

An easy way to compute the total probabilities is taking a uniform two-dimensional grid and computing the ratio of initial conditions that belongs to each basin of attraction. We have done this for different resolutions of the grid as we can see in Fig 5, where it can be clearly observed that the pattern of the basins of attraction is almost stable for resolutions higher than 300 × 300. All the basins of attraction keep their shape near the location of the attractors, but as we move away from them, they begin to mix and become fractal. In Table 1 we summarize the number of initial conditions taken for every resolution and going to each attractor.

**Table 1:** Points per basin.

Phase space size	Precision	Initial points	P3 (Blue)	P1D (Green)	P1I (Red)
50 × 50	0.08	2,500	1,092	700	708
100 × 100	0.04	10,000	4,623	2,687	2,690
200 × 200	0.02	40,000	18,248	10,845	10,907
300 × 300	0.0133	90,000	41,089	24,326	24,585
400 × 400	0.01	160,000	73,159	43,218	43,623
500 × 500	0.008	250,000	114,298	67,780	67,922
1,000 × 1,000	0.004	1,000,000	455,877	270,612	273,511
2,000 × 2,000	0.002	4,000,000	1,826,335	1,081,648	1,092,017
3,000 × 3,000	0.00133	9,000,000	4,104,916	2,434,470	2,460,614

**Fig 6:**
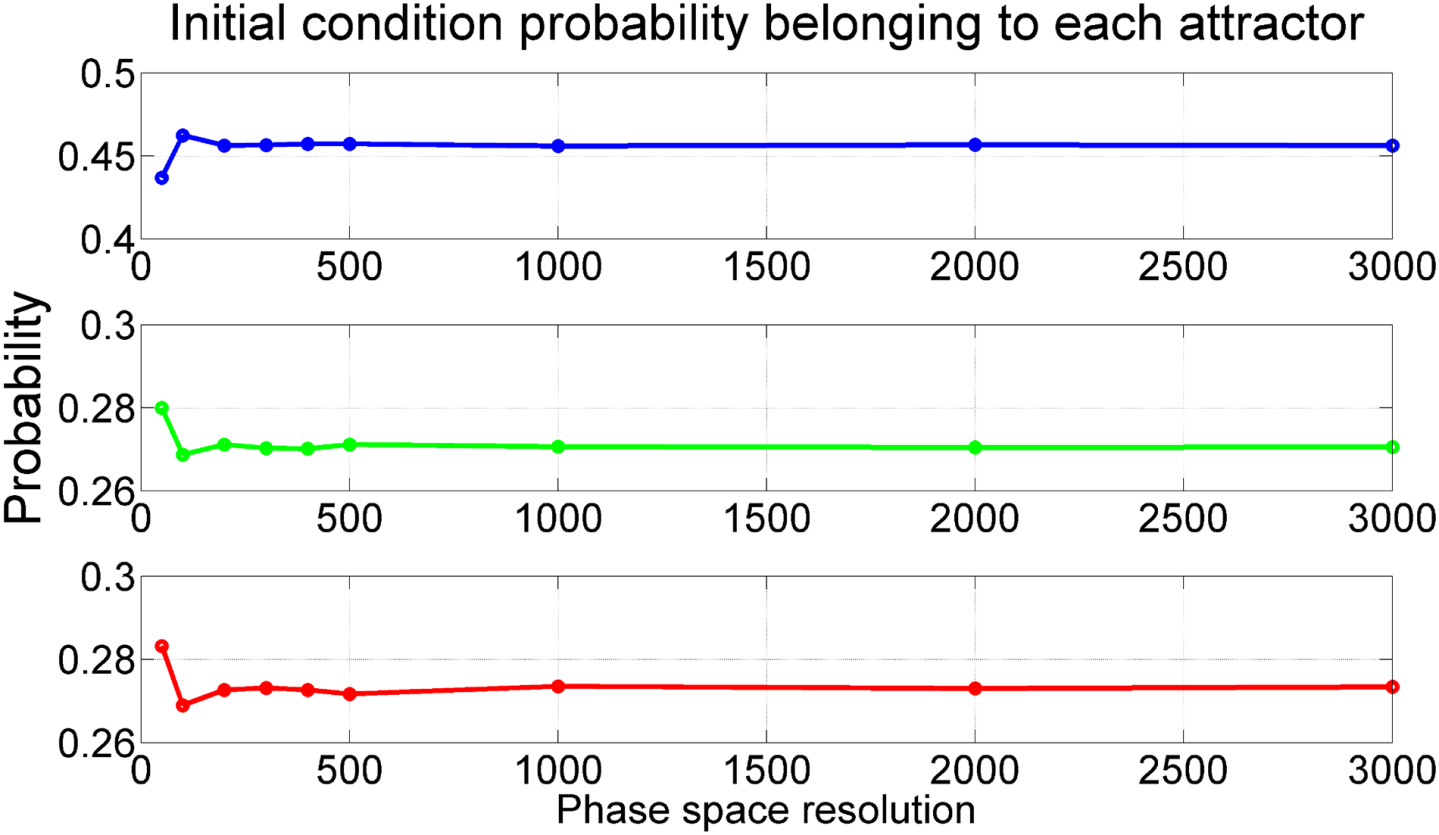
The attractors probability trends. This figure shows the probabilities of each basin of attraction in the phase space (vertical axis) and the resolutions of each phase space given by it matrix size (horizontal axis). The blue line represents the initial condition probability belonging to the period-3 attractor *P*3, the green line corresponds to the period-1 attractor *P*1*R*, and the red line to the period-1 attractor *P*1*L*.

**Fig 7:**
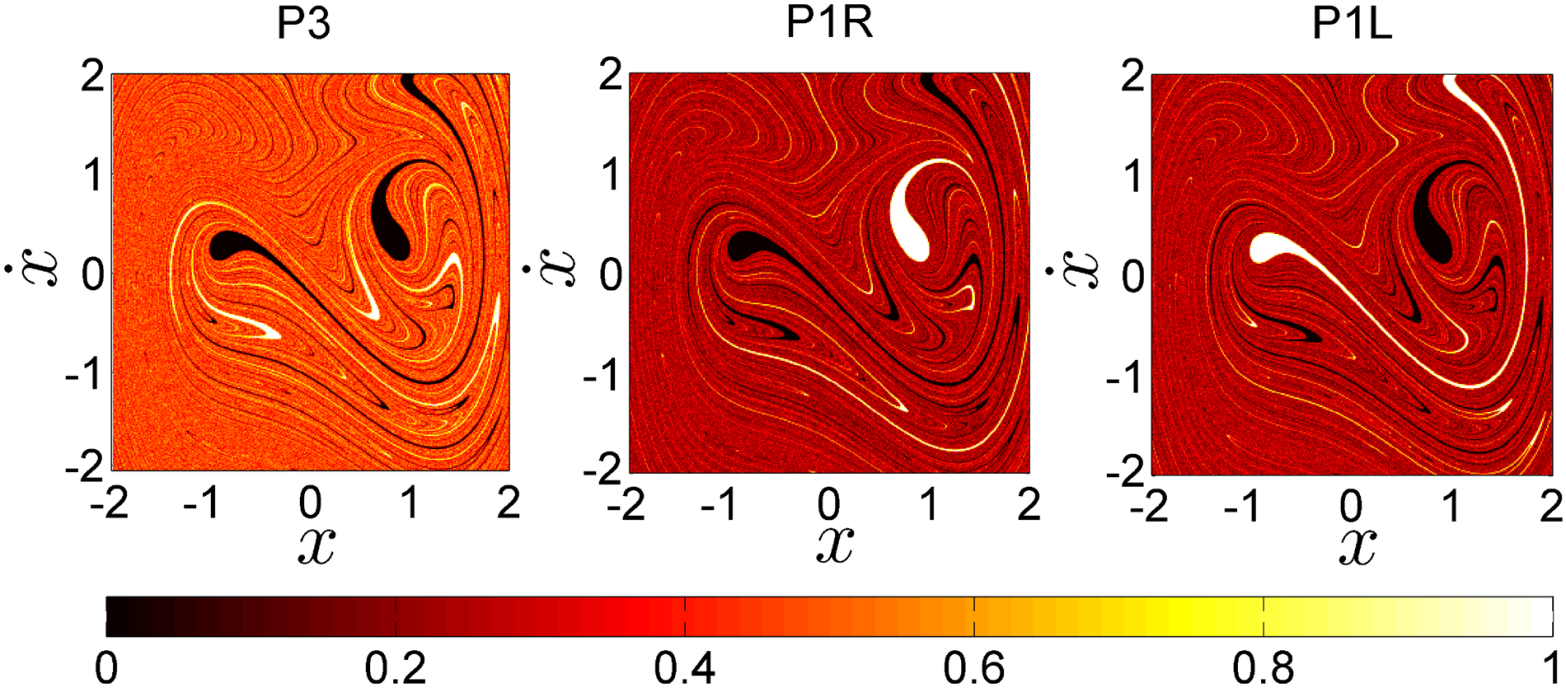
The probability of the attractors on the phase space. We have divided the original phase space into 90, 000 samples squares where each square possesses a sample of 100 grid points from the the original 3000 × 3000 phase space. Then, we have computed the relative frequency of each attractor in each square over the phase space. We have plotted the probability of each attractor on the phase space separately. *P*3 on the left panel, *P*1*R* on the central panel and *P*1*L* on the right panel. When there is 100 percent probability for the square to fall into a particular attractor we have colored the square in white. When there is no chance (probability 0) for a particular square to fall into a particular attractor we have colored the square in black. When the probability of falling into a particular attractor is between 0 and 1 we have colored the square in some red scale color, where dark red is close to probability 0 and yellow is close to probability 1. Note that here we are not plotting the basins of attraction, but the spatial probability function associated with each attractor.

For very low grid resolutions there is a large change in the probabilities going to each attractor. But beyond a given threshold in the resolution, the probabilities remain constant. This is what we show in Fig 6. We can clearly see how, for a resolution of 300 × 300 or higher, the probabilities converge to constant values. The total probability of landing in the period-3 attractor *P*(*P*3) (blue basin) converges to 0.456 (45.6%), the total probability of landing in the period-1 attractor to the right *P*(*P*1*R*) (green basin) converges to 0.270 (27%) and the total probability of ending in the period-1 attractor to the left *P*(*P*1*L*) (red basin) converges to 0.274 (27.4%). This clearly indicates that the results are robust and can be used in the statistical prediction that we are looking for. As expected, due to the convergence of the Perron-Frobenius operator these probabilities are scale free. Fig 7 shows how the probability of each attractor changes depending on its location over the phase space. Now we can actually visualize why and even where the probability of being in the basin of the period-3 attractor, for example, is greatest over the phase space. The orange color on the left panel in Fig 7 illustrates how the high probability of the period-3 attractor dominates in the fractalized zones, while in the other two panels the dark red color illustrates the low probability of the period-1 attractors over the same palaces in the phase space. The fact that the fractal zones occupy a larger area of the phase space explains why at the aggregate level we obtain the results above. We can state that the long term dynamics of this system depends on the attractor that governs in the fractal zones.

Surprisingly, we find here a very remarkable result in the rows and columns with a strong Wada property. In those regions it is satisfied that

$$\begin{array}{c} P\left(P1L|x=x_{i}\right)\approx P\left(P1L|\dot{x}\approx {\dot{x}}_{i}\right)\approx P\left(P1L\right)\\ P\left(P1R|x=x_{i}\right)\approx P\left(P1R|\dot{x}={\dot{x}}_{i}\right)\approx P\left(P1R\right)\\ P\left(P3|x=x_{i}\right)\approx P\left(P3|\dot{x}={\dot{x}}_{i}\right)\approx P\left(P3\right). \end{array}$$ (6)

This means that in the regions with the Wada property the knowledge of one of the coordinates of the initial condition does not improve our prediction capability. It is the same as not knowing any of the coordinates of the initial condition. The conditional probability and the total probability differ only in the regions with large basin cells.

As we have just seen, the probabilities of each basin of attraction converge to a constant probability when the resolution of the phase space increases as shown in Fig 6. It seems that improving the resolution does not affect the probabilities anymore. To show that this result does not have any grid dependence, we have carried out a Monte Carlo simulation. We have chosen the Monte Carlo method because the error on the results typically decreases as $1/\sqrt{N}$ [17].

**Fig 8:**
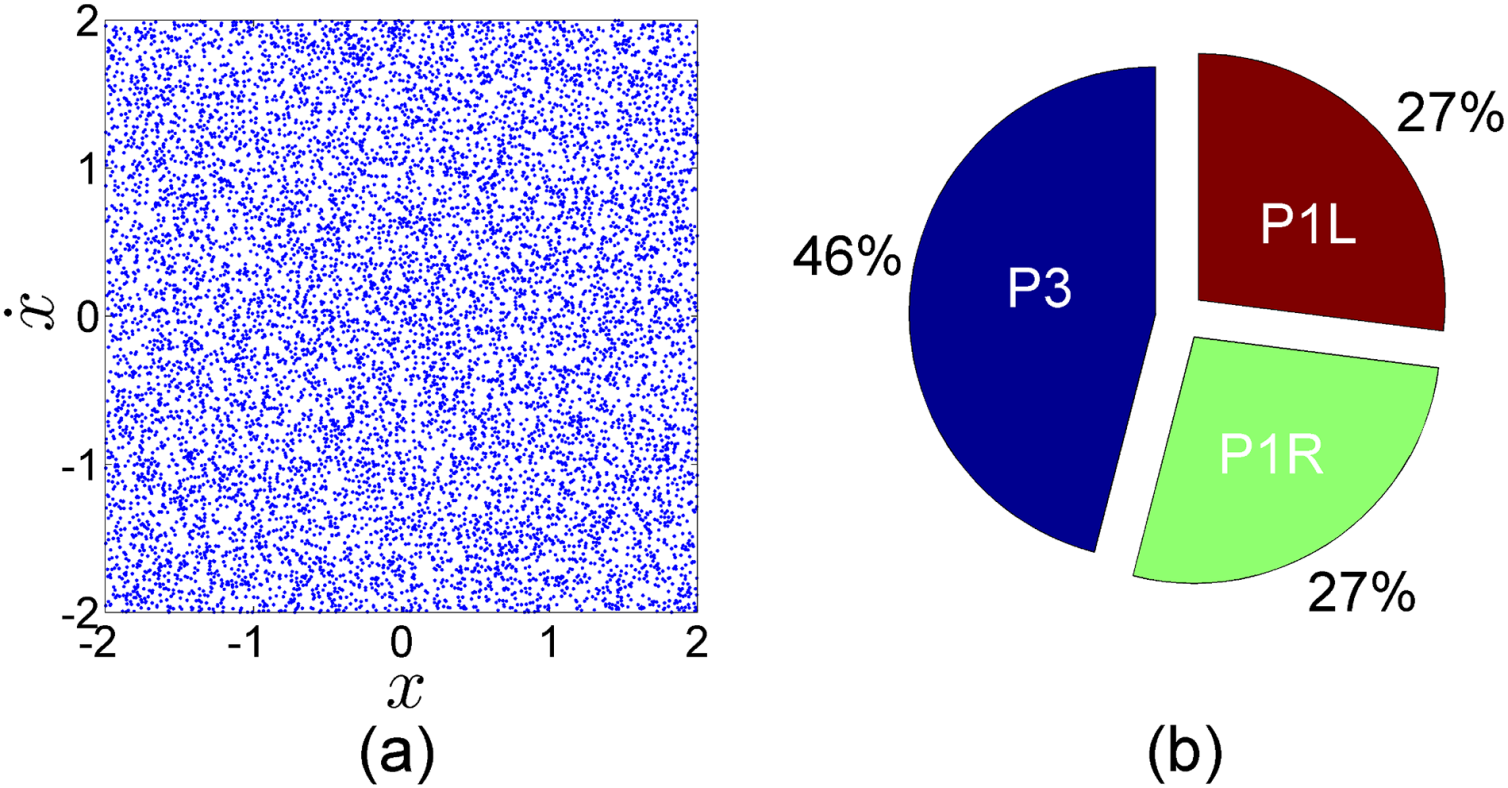
The probability of the attractors when the initial conditions are chosen randomly. (a) Shows the initial conditions taken in the Monte Carlo method to compute the probability associated to each attractor. The pie diagram in (b) shows the probability of each attractor in the phase space according to this sample of initial conditions.

To implement the Monte Carlo method, we have chosen randomly 50, 000 initial conditions with 15 decimals precision in phase space. This precision is equivalent to a fine grid of 10^16^ × 10^16^ initial points. We have used a uniform probability distribution to generate the initial conditions as shown in Fig 8. Then, we have integrated them using a fourth-order Runge-Kutta integrator with a fix integration step of 2*π*/200 and classifying them depending on the attractor towards which they converge. We have chosen bigger integration steps for this calculation because of the high precision of initial conditions. Next, we have computed the ratio of initial conditions going to each attractor versus the total number of initial conditions in the sample to obtain the attractors probabilities. We have obtained the following results; the total probabilities of the period-1 attractors *P*1*R* and *P*1*L* are 0.270 and 0.272 respectively. The total probability of the period-3 attractor is 0.458. These probabilities are almost identical to the probabilities found in the statistical analysis found with the uniform grid. With this result, we can confirm the probabilities obtained with the uniform grid for the statistical predictions of an arbitrary initial condition are very accurate.

## Implication of fractal boundaries on the probabilities of each basin of attraction

There is a very intuitive explanation for the convergence of the total probability of each attractor towards a constant value, as shown in the previous sections. The fractal basins that we study here have a fractal dimension, though we have not computed it since it is not relevant for the statistical predictions that we were studying. Simply by looking at Fig 5 it is clear that we face self similar basins of attraction that do not change with the scale at which they are measured [18]. The method used to compute the probability of each basin of attraction in phase space, is somehow like measuring the area that each basin occupies in the phase space. This is similar to what happens in the famous coastline paradox [19]. As we increase the resolution, the perimeter of the coastline increases towards the infinity. But the area enclosed by that perimeter remains constant [20, 21]. A completely analogous behavior is found in the case of the Duffing oscillator. We get more points on the basin boundaries and the precision of each point increases as well. But the area of each basin of attraction occupies the same space in all scales of the phase space from a given threshold resolution. This behavior is helpful when we are interested in the global dynamics of the system. In some dynamical systems with sensitive dependence on initial conditions, knowing the attractor’s probabilities is enough to understand the system and to do statistical predictions. In many cases, making clever decisions in accordance to the probability of every attractor in the system is good enough.

## Conclusion

In this paper, we have studied the Duffing oscillator model with a choice of parameters showing the Wada property. Then, by using methods from statistical analysis, the probabilities of ending up in a particular attractor of the phase space have been found. We have also shown that these probabilities might be scale invariant. This result is related to the fractal nature of the basins boundaries. A Monte Carlo simulation has been used to verify the values of the attractor probabilities and we have found that are very similar to the values calculated in the statistical analysis. We have shown that knowing the attractors probabilities in some cases is enough to predict the future state of the system and to tackle the final state sensitivity problem, even if we do not have any knowledge about the initial conditions of the system. We have also shown how relatively low grid resolutions (300 × 300 or higher) are enough to obtain the statistical information needed for the statistical predictions about the future state of the system, even in systems as complicated as the Duffing oscillator with the Wada property. This means that we can save a lot of time, effort and memory space when computing the probabilities associated with this kind of systems. Finally, we have also seen how in terms of prediction, the knowledge of one of the coordinates of the initial condition, provides similar results to the case when the two coordinates in the Wada regions are unknown. The technique presented here can be applied to any dynamical system with fractal basins or even Wada basins over its phase space. We believe that using this technique with relative low resolution phase space samples might give a good understanding of the attractors distributions and probabilities helping decision makers and researchers to make decisions and even predict, optimizing their computational time and resources.
